# Purification of Reversibly Oxidized Proteins (PROP) Reveals a Redox Switch Controlling p38 MAP Kinase Activity

**DOI:** 10.1371/journal.pone.0015012

**Published:** 2010-11-15

**Authors:** Dennis J. Templeton, Myo-Sabai Aye, Joshua Rady, Fang Xu, Janet V. Cross

**Affiliations:** Department of Pathology, University of Virginia School of Medicine, Charlottesville, Virginia, United States of America; Auburn University, United States of America

## Abstract

Oxidation of cysteine residues of proteins is emerging as an important means of regulation of signal transduction, particularly of protein kinase function. Tools to detect and quantify cysteine oxidation of proteins have been a limiting factor in understanding the role of cysteine oxidation in signal transduction. As an example, the p38 MAP kinase is activated by several stress-related stimuli that are often accompanied by in vitro generation of hydrogen peroxide. We noted that hydrogen peroxide inhibited p38 activity despite paradoxically increasing the activating phosphorylation of p38. To address the possibility that cysteine oxidation may provide a negative regulatory effect on p38 activity, we developed a biochemical assay to detect reversible cysteine oxidation in intact cells. This procedure, PROP, demonstrated in vivo oxidation of p38 in response to hydrogen peroxide and also to the natural inflammatory lipid prostaglandin J2. Mutagenesis of the potential target cysteines showed that oxidation occurred preferentially on residues near the surface of the p38 molecule. Cysteine oxidation thus controls a functional redox switch regulating the intensity or duration of p38 activity that would not be revealed by immunodetection of phosphoprotein commonly interpreted as reflective of p38 activity.

## Introduction

Cell signal transduction is a summation of positive stimuli and homeostatic negative feedback. Positive regulation of protein kinase cascades, such as those activated during mitogenic signaling, differentiation, and pathogenic processes such as inflammation and cancer, is particularly well studied. Most commonly, activation of a kinase cascade results from sequential phosphorylation of protein kinases by other kinases positioned upstream. In contrast, other than the action of phosphatases that remove the activating phosphorylation, inhibitory or negative feedback mechanisms for damping or silencing protein kinase cascades are less well understood. In fact, mainly due to the utility of anti-phosphoepitope antibodies in identifying phosphate on specific proteins, it has become common in the literature for the presence of an activating phosphorylation on a signaling kinase to be interpreted as an ‘active’ kinase pathway. This unfortunate simplification ignores more subtle means of signal regulation.

Many signaling events, including UV irradiation, inflammatory cytokines, and mitogen stimulation, are accompanied by generation of reactive oxygen species [1], [2]. These oxidative species include those that acquire one electron from NAD(P)H and those that acquire two electrons from NAD(P)H [3]. One-electron oxidoreduction reactions result in the release of high-energy species generated by the unused electron from NAD(P)H. These species include superoxide, hydroxyl radical, and others, that likely contribute to damaging modifications of proteins and lipids. Two-electron oxidoreduction mainly results in the in situ generation of hydrogen peroxide, that can be used by endogenous enzymes to oxidize cysteine residues on proteins. These modifications take several forms, including oxidation to sulfenic acid (R-SOH) or formation of disulfides involving intra- and intermolecular connections between proteins or S-thiolation of proteins by glutathione and cysteine. Reversible oxidation of proteins on cysteines is potentially a means for controlling signal transduction, since it adds an often-charged, bulky moiety to the protein primary structure, and is reversible, much like protein modifications resulting from phosphorylation [4], [5]. For example, the activity of the MEKK1 protein kinase is regulated by reversible glutathionylation of a single cysteine residue in the ATP binding pocket [6]. Several other kinases have been shown to be activated or inhibited by reversible cysteine oxidation [7], [8], [9], [10], [11], [12], [13], [14], [15], though mainly with non-physiologic stimuli.

Compared to the study of protein phosphorylation, study of specific oxidation on proteins is difficult. Antibodies against S-glutathionylation have been described, and protein sulfhydryls have been labeled with fluorescent maleimides or iodoacetamides. We have developed an unbiased method of quantifying reversible cysteine oxidation in proteins using an affinity capture technique we call Purification of Reversibly Oxidized Proteins (PROP). We have applied this technique to study the regulatory oxidation of the p38 MAPK signaling kinase. We have found that p38 is oxidized following exposure of cells to exogenous hydrogen peroxide or prostaglandin J2, and that oxidation results in kinase inactivation despite continued phosphorylation on the activating residues detected by immunoblot. Prostaglandin J2 (PGJ2) is an inflammatory mediator that has been characterized for its role in inducing oxidative signals [16], [17]. As such, the oxidation of p38 following exposure of cells to PGJ2 reflects a relevant physiologic signaling event that might occur during inflammation. The PROP procedure provides a facile means to quantify oxidation of known protein targets and to identify new targets of potential regulatory oxidation events.

## Results

### Inhibition of p38 activity by protein oxidation

**Figure 1:**
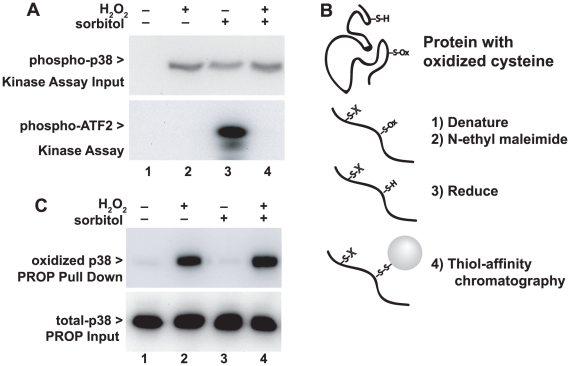
Thiol oxidation and inhibition of p38. Panel A. Hydrogen peroxide inhibits p38 kinase activity but not phosphorylation. Thirty minutes before harvest, HeLa cells grown in 6-well plates were treated with 10 mM H_2_O_2_. Ten minutes later, some wells were treated with 0.4 M sorbitol to activate p38 through osmotic shock. (top) Both H_2_O_2_ and sorbitol induced phosphorylation of p38. (bottom) P38 activity measured with an in vitro kinase activity was only present in osmotically-stimulated cells not treated with hydrogen peroxide. Kinase activity was not correlated with phosphorylation of p38. The figure is representative of five independent experiments. Panel B. Outline of the PROP procedure to purify reversibly thiol-oxidized proteins (see details in Methods). Cultured cells are rapid fixed in TCA, then proteins denatured and solubilized in guanidine buffer containing NEM to block non-oxidized thiol groups on proteins. Oxidized proteins are reduced with DTT, then proteins with newly revealed free thiols isolated using thiol-affinity chromatography. Proteins are released from the affinity beads and subjected to SDS-PAGE and immunoblotting, compared to an aliquot of total proteins taken at step 1. Panel C. Hydrogen peroxide treatment results in reversible thiol oxidation of p38 detected using PROP. HeLa cells treated as in Panel A were subjected to PROP. (top) Reversible oxidation of p38 was detected in cells treated with 10 mM H_2_O_2_ but not in untreated cells. (bottom) Treatments did not alter the abundance of p38 in total lysates. The figure is representative of over 20 experiments.

To test the effect of oxidation on p38 kinase activity, we treated cells with hyperosmotic sorbitol to activate p38, with 10 mM H_2_O_2_, or both. As expected, we found that both osmotic shock and hydrogen peroxide, or both, induced phosphorylation of p38 detected with anti-phospho-p38 antibodies (Figure 1A, top). We then tested the in vitro kinase activity of p38 by using immune complexes containing p38 from aliquots from these same cells, measuring phosphorylation of the p38 substrate ATF2 (Figure 1A, bottom). Osmotic shock induced p38 kinase activity as expected, but H_2_O_2_ treatment did not induce kinase activity despite strongly inducing p38 phosphorylation (lane 2). Additionally, H_2_O_2_ treatment of osmotically-shocked cells completely extinguished the p38 kinase activity, despite having no effect on p38 phosphorylation (lane 4). These results suggests that H_2_O_2_ blocks p38 kinase activity through a mechanism that does not decrease activating phosphorylation, probably cysteine oxidation.

**Figure 2:**
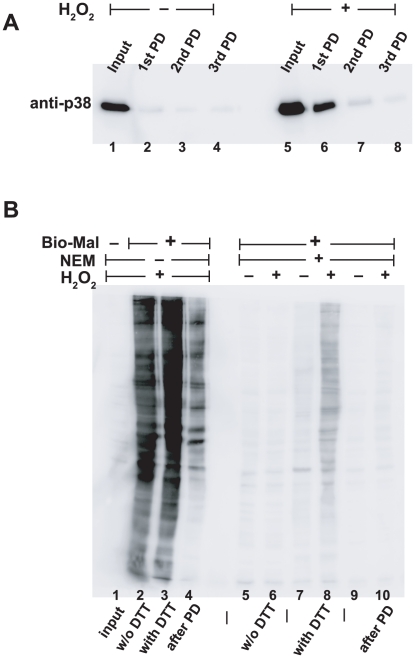
Validation of efficiency and specificity of PROP procedure. A. Specificity and efficiency of activated thiol bead purification. Extracts containing unoxidized p38 (left) or p38 from cells oxidized with 10 mM H_2_O_2_ (right) were sequentially incubated with activated thiol beads after PROP (1st, 2nd, or 3rd PD respectively). Compared to the starting material (“input”) little p38 was recovered on the beads from the untreated, unoxidized sample. In contrast, the 1st precipitation of the oxidized p38 recovered much of the input p38, while little remained in solution that could be precipitated in subsequent repeat precipitations. B. Efficiency of sulfhydryl blockade using NEM. HeLa cells treated with 10 mM H_2_O_2_ were processed through PROP, with equal aliquots taken at several points along the procedure. Proteins were treated with Biotin-maleimide to label free sulfhydryls that had escaped NEM blockade, and biotin was detected with anti-biotin immunoblot.

To test this possibility, we devised a method to quantify oxidation of specific proteins. We developed and optimized a procedure for Purification of Reversibly Oxidized Proteins (PROP) described in detail in Methods. This procedure is outlined in Figure 1B. Briefly, cell metabolism is stopped in strong acid, then cell proteins are dissolved in guanidine denaturing buffer. Reduced protein thiols are blocked by exhaustive treatment with N-ethylmaleimide, then the NEM is inactivated and protein reduction effected by treatment with an excess of dithiothreitol (DTT) at elevated temperatures. DTT is known to reverse sulfenic acid and S-nitrosylation, as well as most disulfide bonds. Proteins are recovered through precipitation, and the newly-reduced thiols resulting from the originally oxidized sites are recovered by a commercial preparation of activated thiopropyl agarose beads, that contain a dipyridyl disulfide group that forms spontaneous disulfides with free thiols. The originally-oxidized proteins are bound to the activated beads and recovered, washed and eluted in DTT-containing sample buffer for immunoblot analysis. Summarizing briefly, the procedure efficiently blocks unoxidized cysteines, and efficiently recovers proteins that were initially oxidized. Validation of the PROP procedure is described below in conjunction with Figure 2.

We suspected that H_2_O_2_ induced oxidation of one or more cysteine residues on p38. We used the PROP procedure to measure reversible oxidation on p38. HeLa cells treated as above with hyperosmotic sorbitol and/or H_2_O_2_ were subjected to the PROP procedure, and the recovered proteins analyzed for p38 (Figure 1C, top). Immunoblotting for p38 revealed recovery of p38 in the PROP pull-down of the H_2_O_2_ treated samples, reflecting reversible p38 oxidation in cells treated with H_2_O_2_, whether or not the cells were osmotically shocked. Minimal oxidized p38 was detected in untreated cells or osmotically shocked cells without H_2_O_2_. Equal expression of p38 in all samples was confirmed by immunoblotting of an aliquot of the total cell lysates (Figure 1C, bottom).

### Validation of the PROP procedure

Extracts containing p38 oxidized by H_2_O_2_ were used to validate the PROP procedure. We first tested the efficiency of recovery of oxidized proteins using the thiol bead pull down (Figure 2A). Using either detergent extracts of unoxidized cells (four left lanes) or extracts of cells oxidized with 10 mM H_2_O_2_ to oxidize p38 (four right lanes), an aliquot of the input sample was analyzed adjacent to equivalent fractions of serial precipitations using the activated thiol sepharose beads. Essentially no p38 was precipitated from the unoxidized cell extract (lanes 2, 3, and 4). Oxidized p38 was recovered in the first pull down from the oxidize cells (lane 6) but not from the repeatedly precipitated extracts (lanes 7 and 8). Thus, the precipitation of oxidized proteins does not recover unoxidized proteins, and a single precipitation is essentially complete.

Next, the efficiency of blocking of unoxidized proteins using NEM in the PROP procedure was tested. In the absence of blocking NEM (Figure 2b, lanes 1–4), unblocked thiols were readily detected using biotin-maleimide and detection of biotin label using anti-biotin immunoblotting (lane 2). No anti-biotin signal was evident without the biotin-maleimide treatment (lane 1). Biotin-maleimide labeling increased when extracts were treated with DTT to reduce oxidized thiols (lane3). The majority of proteins that could be labeled with biotin-maleimide were removed using activated thiol beads (lane 4). Importantly, the number of free protein thiols in this reaction is far higher than in the precipitation step used in the actual PROP procedure because of the lack of NEM blockade; thus this inefficiency does not reflect the efficiency of precipitation documented in Panel A.

Compared to these unblocked samples, NEM blockade of thiols in oxidized or unoxidized samples was greatly diminished (lanes 5 and 6); thus NEM blockade of free thiols is efficient. As predicted labeling with biotin-maleimide could be increased in the oxidized cell sample using DTT (lane 8), and less so in unoxidized cells (lane 7). Following precipitation of these cell extracts using activated thiol beads (lanes 9 and 10) essentially no proteins were labeled with biotin-maleimide. Thus, again thiol bead precipitation using activated thiol beads efficiently removed the initially-oxidized proteins from cell extracts.

### Hydrogen peroxide blocks p38 activity in intact cells

**Figure 3:**
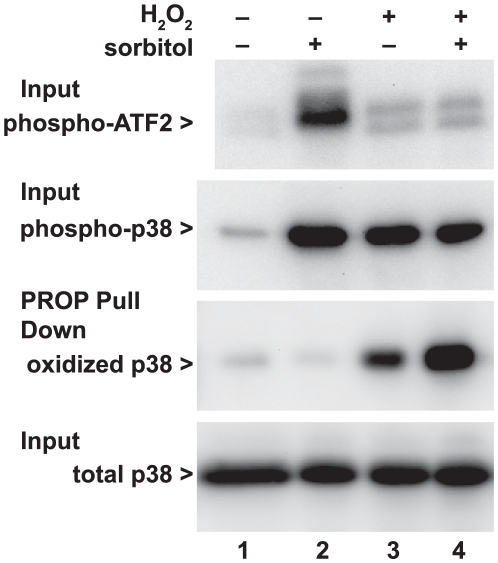
Oxidation by H_2_O_2_ inhibits p38 kinase activity in intact cells. HeLa cells were seeded in 6-well plates. At 30 min prior to harvesting, the indicated wells were treated with 10 mM H_2_O_2_ and 10 min later, the indicated wells were treated with hyperosmotic 0.4 M sorbitol to activate p38. PROP pulled down oxidized p38 only after H_2_O_2_ treatment (lanes 3 and 4). Both sorbitol and H_2_O_2_ induced phosphorylation of endogenous p38 (lanes 2,3,4). However, while endogenous phosphorylated ATF-2, an in-vivo substrate of p38, was seen after activation of p38 with hyperosmotic sorbitol (lane 2) phosphorylation of ATF-2 was absent in cells treated with both hyperosmotic sorbitol and H_2_O_2_. Thus, in vivo activity of p38 (indicated by phosphorylation of the p38 substrate, ATF-2) did not correlate with the conventional measure of p38 activity, i.e. phosphorylation of p38. The amount of endogenous p38 following any treatment (bottom panel) was unchanged. The figure is representative of two independent experiments.

While oxidation of cells reduced the activity of p38 measured in *in vitro* assays, we sought to confirm that this reflected the activity of p38 in intact cells. We repeated the experiment shown in Figure 1, activating cells with hyperosmotic sorbitol to activate p38, and subsequently treating cells with hydrogen peroxide to inhibit p38 (Figure 3). Again, p38 phosphorylation (second panel) was strongly activated by both hyperosmotic sorbitol or hydrogen peroxide, or both. However, immunoblotting of the phopshorylated p38 substrate ATF2 (top panel) showed the intact 38-ATF2 axis was blocked in cells treated with H_2_O_2_. Again PROP revealed oxidation of p38 by H_2_O_2_ treatment (bottom two panels).

### The natural cell oxidant prostaglandin J2 also regulates p38 oxidation and activity

Since 10 mM H_2_O_2_ is a super-physiological oxidative stimulus, we tested the natural prostaglandin, PGJ2, for oxidation on p38. PGJ2 is an arachidonic acid metabolite associated with inflammation that has been implicated in regulatory oxidation of other proteins, for example p53 [16], [17].

**Figure 4:**
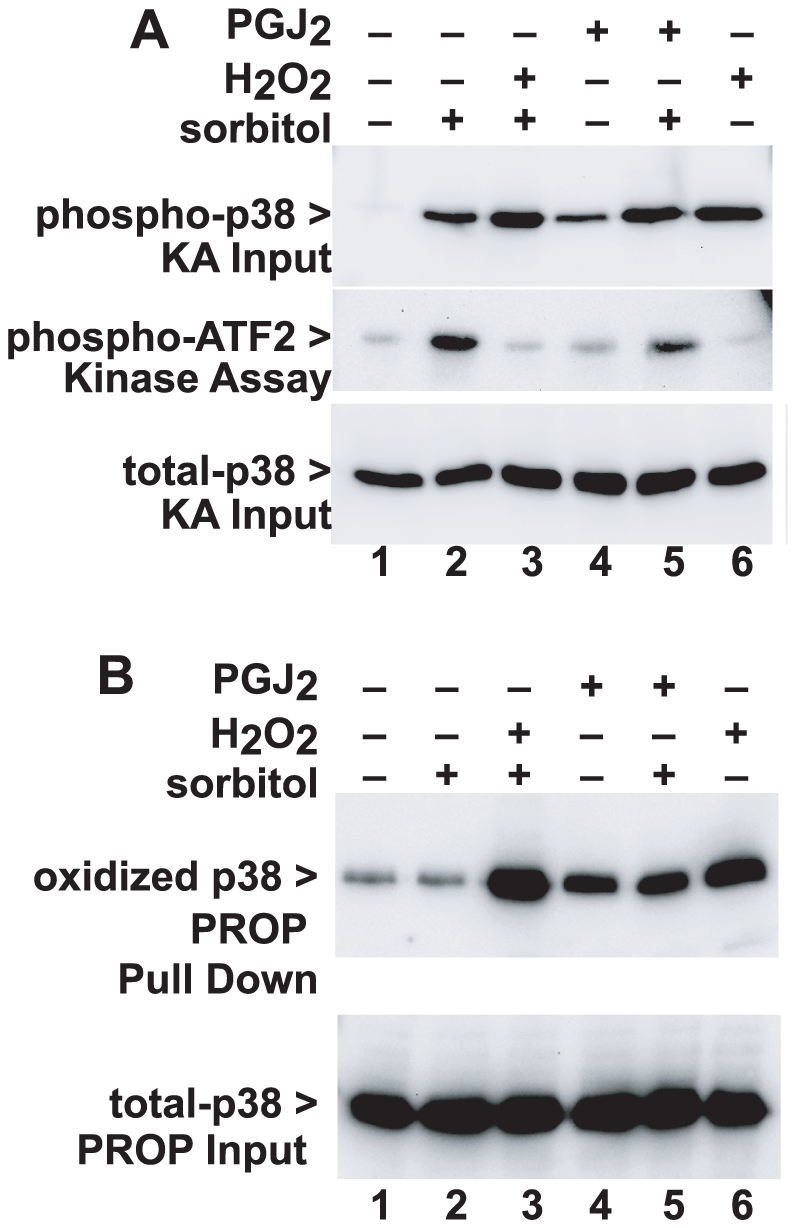
The natural oxidant prostaglandin J2 induces oxidation of p38 and inhibits activity. Panel A. HeLa cells in 35 mm wells were treated with 100 µM prostaglandin J2 (PGJ2) for 1 hr or 10 mM H_2_O_2_ for 30 min as indicated. Some wells were treated 20 min prior to harvesting with hyperosmotic sorbitol to activate p38. Whole cell lysates (top) showed phosphorylation of p38 by both oxidants (lanes 3–6) and by osmotic shock alone (lane 2). In vitro kinase activity was detected (middle) only in the wells osmotically shocked with sorbitol, and activation by sorbitol was completely inhibited by H_2_O_2_ (lane 3, **p = 0.0004**) and partially inhibited by PGJ2 (lane 5, **p = 0.004**). Expression of p38 was equivalent in all samples (bottom). The figure is representative of five independent experiments. Panel B. PROP analysis of the lysates made for the experiment in Panel A (top) showed oxidation of p38 induced by both PGJ2 (lanes 4 and 5) and H_2_O_2_ (lanes 3 and 6) irrespective of osmotic shock. The degree of oxidation detected correlated with the degree to which p38 activity was inhibited, in Panel A. Expression of p38 was unchanged in all samples (bottom). The figure is representative of four independent experiments.

Hyperosmotic sorbitol, H_2_O_2_, and PGJ2, all induced p38 phosphorylation (Figure 4, Panel A, top). As before, H_2_O_2_ completely blocked p38 kinase activity induced by hyperosmotic shock, as measured by in vitro phosphorylation of ATF2 substrate (Figure 4A, middle, compare lanes 2 and 3), while 100 µM PGJ2 reduced p38 activity by 38% (compare lanes 2 and 5). These results were reproducible over five independent experiments; sorbitol activation of p38 was inhibited by both H_2_O_2_ (71.5+/−21.6% inhibition, p = .0004) and PGJ2 (37.3+/−11.5% inhibition, p = .004).

Both H_2_O_2_ and PGJ2 induced p38 oxidation, as revealed by PROP (Figure 4B). PGJ2 induced 23% of the amount of oxidation induced by H_2_O_2_ (Figure 4B, top, lanes 3 and 5), consistent with the fractional reduction in p38 kinase activity. Sorbitol treatment alone did not cause p38 oxidation. Thus, quantifying and comparing p38 phosphorylation and kinase activity shows a discordance between anti-phospho-p38 signal and actual kinase activity. For both H_2_O_2_ and PGJ2, inhibition of p38 kinase activity paralleled the degree of oxidation observed by PROP.

**Figure 5:**
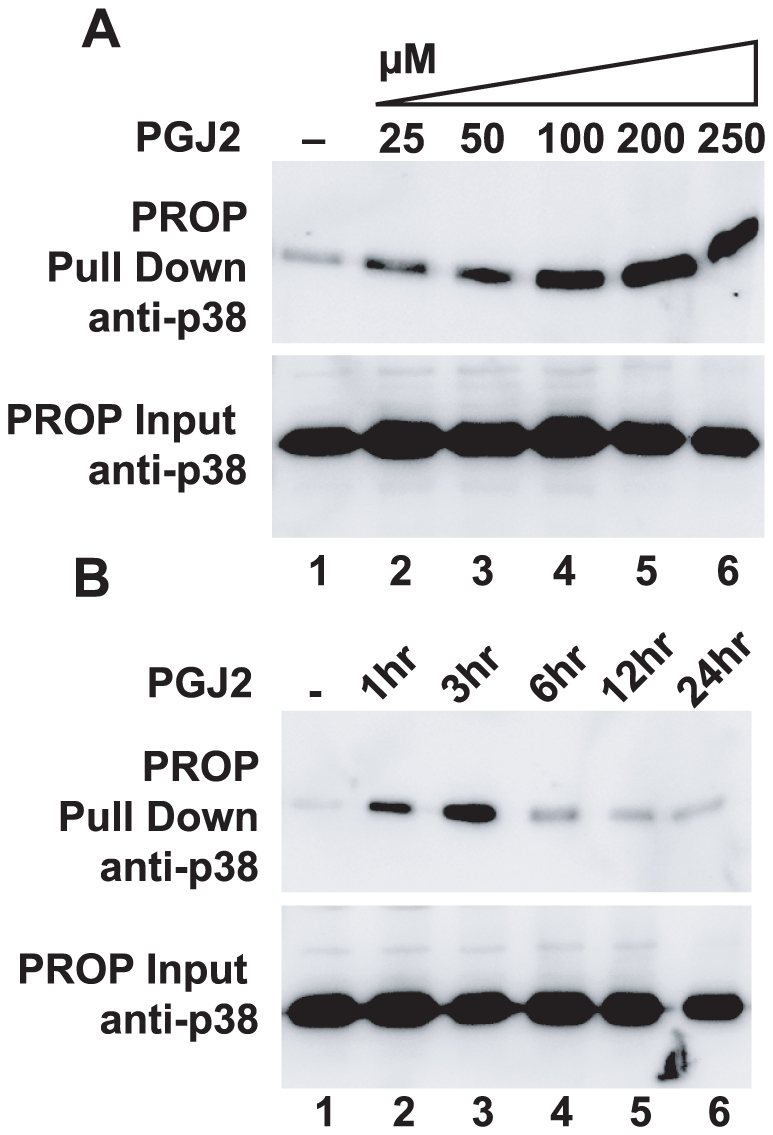
Prostaglandin J2 induces p38 oxidation in a dose and time dependent manner. Panel A. HeLa cells grown in 35 mm wells were treated with 25, 50, 100, 200, or 250 µM PGJ2 for 1 hr before lysis. PROP analysis showed increasing oxidation of p38 with PGJ2 concentration. Panel B. HeLa cells as above treated with 100 µM PGJ2 for 1, 3, 6, 12, or 24 hrs showed oxidation of p38 appearing at 1 hour and increasing at 3 hours. At 6 hours and beyond little oxidation of p38 was detected. The figure is representative of two independent experiments.

The induction of p38 oxidation by PGJ2 was dose responsive (Figure 5A), with distinct oxidation detected with PROP at the lowest dose tested, 25 µM. Oxidation was maximal at the 100–200 µM range. P38 oxidation induced by 100 µM PGJ2 was also time dependent, with reversible oxidation detected at 1 hour after treatment, and increasing at the 3 hour time point (Figure 5B). Reversible oxidation of p38 was no longer detected at 6 hours or subsequent time points. Time dependence was not observed using 10 mM H_2_O_2_, which rapidly oxidized and inhibited p38 (data not shown).

### Oxidized, inactive p38 can be reactivated by reduction in vitro

**Figure 6:**
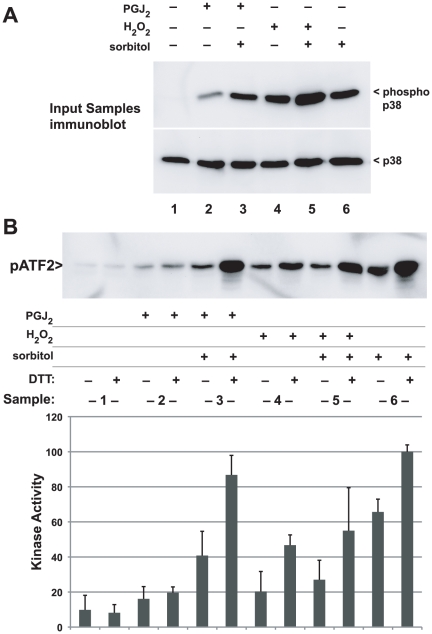
DTT reactivates p38 following inhibition by oxidation. HeLa cells grown in 35 mm wells were treated with 100 µM PGJ2 for 1 hr or 10 mM H_2_O_2_ for 30 min. At 20 min prior to harvest, the indicated wells were treated with 0.4 M sorbitol to activate p38. In vitro kinase assays measuring phosphorylation of ATF2 were performed. Samples were divided in two and immune complexes containing p38 treated in vitro with 10 mM DTT for 10 minutes at room temperature. Panel A. Samples prepared for the reactivation experiment showed activating phosphorylation of p38 (top) and equal expression total p38 (bottom panel) similar to Figure 2. Panel B. In vitro kinase assay showed that DTT did not activate the unstimulated p38 (Sample 1, negative control) while DTT treatment did increase the already strong activity resulting from hyperosmotic sorbitol treatment (Sample 6, positive control). Sorbitol-stimulated p38 activity was inhibited by both H_2_O_2_ (compare Samples 6 and 5, without DTT, p = 0.007) and PGJ2 (compare Samples 6 and 3 without DTT, p = 0.05). DTT treatment completely restored PGJ2-treated, sorbitol stimulated p38 (Sample 3, compare to sample 6) and partially restored p38 inhibited by H_2_O_2_ (Sample 5). The immunoblot (top) is representative of three independent experiments used for the quantification (bottom).

We hypothesized that the lack of p38 activity recovered from cells treated with H_2_O_2_ or PGJ2 was the result of reversible oxidation of p38, as detected using the PROP procedure. To test this hypothesis, cells were treated with combinations of sorbitol, H_2_O_2_, or PGJ2 as before. Aliquots of these samples were immunoblotted to assess p38 expression and phosphorylation (Figure 6A), and the remaining lysate used for in vitro p38 kinase assay. We divided the bead-bound immune complexes into two aliquots before assay; one aliquot was treated with 10 mM DTT at room temperature for 30 minutes, while the other aliquot did not receive DTT. In vitro kinase assay was performed using phosphorylation of the ATF2 substrate as the readout (Figure 6B).

DTT treatment of p38 from untreated cells did not increase activity (sample 1), and had only minor effects on the mildly activated p38 from cells treated with PGJ2 alone (sample 2). However, DTT treatment of p38 from cells treated with both sorbitol and PGJ2 (sample 3) strongly (2.5 fold) activated p38 compared to the p38 that was not treated with DTT. In fact, DTT restored 104% of the p38 activity from the PGJ2/sorbitol sample (sample 3) compared to DTT-treated p38 from cells treated with sorbitol but no oxidants (sample 6). Thus, in vitro exposure to DTT completely restores the activity of p38 inhibited by PGJ2.

DTT treatment also increased the activity of p38 69% from cells treated with sorbitol alone (sample 6), suggesting that a fraction of phosphorylated p38 remains inactive as a kinase owing to oxidation in cells not exposed to deliberate oxidants, and perhaps that oxidation is a normal consequence of kinase activation. If so, this is not detected in the PROP assay (Figure 2b, lane 2). It may be that oxidation is restricted to the small fraction of p38 that is activated by sorbitol treatment. While DTT treatment also increased activity of p38 recovered from cells treated with H_2_O_2_ alone (sample 4) or the combination of H_2_O_2_ and sorbitol (sample 5), the amount of activity recovered was reduced (66%) compared to the activity recovered from cells treated with sorbitol alone or sorbitol with PGJ2. Thus, this concentration of H_2_O_2_ may induce partially non-reversible inhibitory oxidation of p38.

### Mapping of p38 oxidation sites

**Figure 7:**
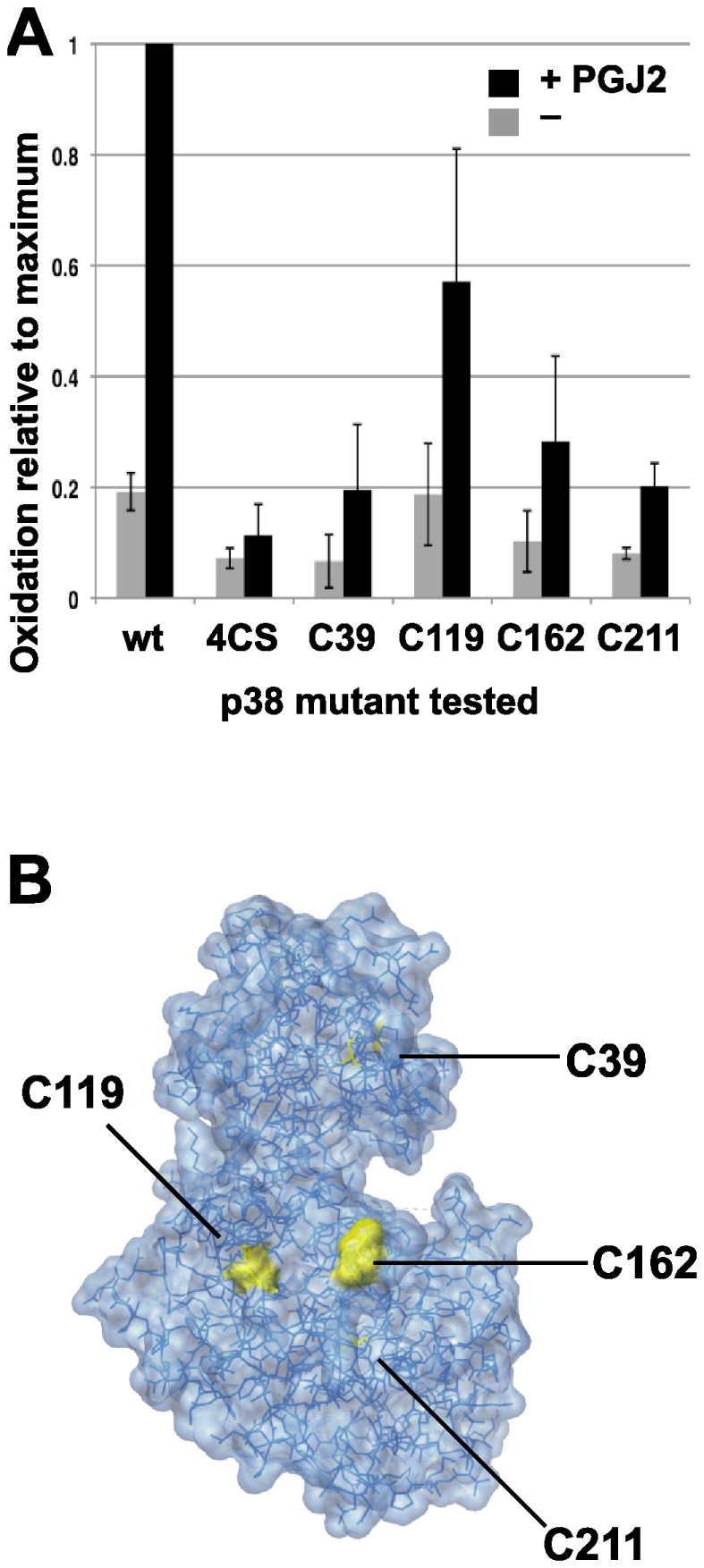
Oxidation of individual cysteines in p38. Plasmids expressing p38 with a carboxy-terminal epitope tag were constructed that expressed otherwise normal p38 (WT), a mutant lacking all four cysteines (4CS), or mutants that contain only one cysteine residue added back to the 4CS mutant (C39, C119, C162, and C211). The encoded proteins were expressed in HeLa cells that were treated with vehicle or with 100 µM PGJ2 for one hour (black bars), and subjected to PROP. Panel A. Oxidation of each mutant protein was determined using anti-p38 immunoblotting. Oxidized fractions are normalized to expression of each mutant protein and oxidation of endogenous p38 in each culture. The proportion of each mutant oxidized is presented relative to the amount of WT p38 oxidation in the PGJ2 treated sample. The data are the average of three completely independent experiments. Panel B. Location of cysteine residues within the p38 protein. Cysteine residues C119 and C162 are located near the surface of the protein, while C39 and C211 are buried within the protein. Structure from PDB, reference ID 1WFC.

The p38 protein kinase has 4 cysteine residues, none of which are predicted to be disulfide bonded. We constructed mutants of p38 containing cysteine-to-serine substitution of all 4 cysteine residues (C4S), four mutants in which each individual cysteine is mutated to serine, as well as four mutants in which single cysteines were added back to the 4CS mutant individually. We tested proteins encoded by each of these mutants for reversible oxidation induced by PGJ2, using PROP. Each of the mutant p38 proteins with individual cysteine to serine substitutions was still oxidized after exposure of cells to PGJ2 as measured by PROP, suggesting that oxidation occurred on more than one cysteine residue (data not shown). Analysis of the mutant p38 proteins in which individual cysteines were added back to the 4CS mutant allowed us to assess the oxidation at each cysteine residue after PGJ2 treatment. Summary results averaging three experiments are shown in Figure 7a. Oxidation of the 4CS mutant protein was not detected, as expected. The mutants with only individual cysteine residues retained weak or strong oxidation, depending on the cysteine residue, with C119 and C162 oxidized to an extent greater than residues C211 and C39. Thus, to the extent that these variant proteins reflect the activities of the natural p38, we conclude that the major sites of reversible oxidation are C119 and C162. The mutant proteins with only a single cysteine residue did not retain catalytic activity, so it was not possible to confirm the anticipated effects on activity using these mutants.

The structure of p38 has been determined [18], and is available in the Protein Data Bank (http://www.pdb.org/pdb/explore/explore.do?structureId=1WFC). This structure confirms that p38 does not have disulfide bonds. It also shows (Figure 7B) that cysteines C119 and C162 lie topologically adjacent to each other near the surface of the protein, while the other two cysteines are buried within the protein matrix. This suggests that these two sites would be more accessible for oxidation following exposure of the cells to PGJ2. Importantly, the migration of p38 from oxidant treated cells on non-reducing SDS-PAGE was similar to that of p38 from untreated cells, and to p38 following reduction with DTT (data not shown), suggesting that intramolecular disulfide bonding or disulfide bridges to another protein is likely not involved in p38 inhibition.

## Discussion

We have developed a new procedure, PROP, to detect and quantify oxidation of cell proteins, and have used it to detect oxidation and inhibition of the stress-activated p38 kinase resulting from both H_2_O_2_ and from the physiologic mediator of inflammation, prostaglandin J2. The PROP assay is similar in concept to the Jaffrey assay for protein nitrosothiols [19], in that protein thiols are first blocked with thiol-reactive reagents, and then modified thiols revealed with DTT (or ascorbate, for nitrosocysteine) before recovery using thiol affinity methods. PROP may have some benefit over this prior assay because it involves fewer steps (no chemical modification of the target cysteines are required for recovery, instead direct thiol affinity is achieved using a one-step commercial reagent). While the Jaffrey procedure blocks thiols in SDS solutions, we found in pilot studies that the use of guanidine instead of SDS as a denaturant resulted in superior blocking of non-oxidized cysteines. Precipitation of guanidine-denatured protein by methanol is an efficient way of recovering protein while removing small cysteine-modifying chemicals including NEM and DTT.

In addition to the Jaffrey method, there are several other approaches for labeling and detecting reversible oxidation of protein targets. Most of these rely on a similar strategy of blocking unmodified cysteines, then reversing the modification and labeling with a thiol reactive reagent that provides a means of detection. Among the available options are fluorescent labels, biotinylated agents, mobility shift, and radioactive compounds [20], [21], [22], [23], [24], [25], [26]. These approaches each have limitations that the PROP procedure obviates. For example, while fluorescent or radioisotopic labeling can enable detection of labeled proteins, these approaches do not purify the labeled (oxidized) proteins away from non-oxidized proteins, that is necessary for proteomics analysis to characterize unidentified proteins. Biotin labeling (as in the Jaffrey or biotin switch method) enables a somewhat efficient purification approach. However, the biotin label on the purified protein can be problematic for mass spectroscopy identification of the modified residue on the oxidized proteins. In the PROP procedure, proteins are released from the thiol beads with reducing agent (e.g. DTT, TCEP etc) in an unmodified form, leaving them completely compatible with downstream applications such as mass spectrometry, or for isotopic labeling such as with maleimides containing heavy isotopes. Alternate methods of protein release with ascorbic acid or arsenite, can be used to elute proteins with specific types of cysteine oxidations. Some biotin-switch methods rely on labeling of cell lysates, which introduces the variability of post-lysis oxidation, despite precautions of cell lysate processing in an anaerobic chamber [27]. Our approach uses rapid fixation of cells using 10% trichloroacetic acid, which immediately prevents post-lytic oxidation. As the acid is removed, proteins are continuously in the presence of thiol-blocking maleimide.

A seemingly simple aspect of the procedure that provides a major advantage is the use of guanidine as a denaturing agent. Guanidine denaturation provided superior blocking of free sulfhydryls when compared to any other approach we attempted, allowing sensitive detection of very small amounts of oxidized protein due to the absence of background labeling that results from inefficient blocking. Removing guanidine can be troublesome, but our use of methanol precipitation of proteins efficiently removes the denaturant, the reductant, and the blocking reagent simultaneously. The resulting protein precipitate is soluble only in strong denaturants (in our case the same guanidine buffer as used for lysis) but this reagent is suitable for use with the activated thiol agarose beads.

Protein thiols are subject to several levels of oxidation. Thiols can form disulfides through interaction with other protein thiols or with small thiols such as cysteine or glutathione. Alternately, thiol oxidation can lead to sulfenic acid (R-SOH) or higher-order sulfinic (R-SO_2_H) or sulfonic (R-SO_3_H) acids. Additionally, nitroso-thiols can result from endogenous or exogenous nitric oxide. Disulfides, nitroso-thiols, and sulfenic acid are non-enzymatically converted to thiols in the presence of reducing agents such as glutathione or DTT, and are certainly detected using the PROP procedure in which modified proteins are subjected DTT. These modifications are likely also reversible in vivo and therefore represent potential regulatory events. Higher order oxidations (sulfinic and sulfonic) are unlikely to be reversed by DTT treatment during PROP. Based on our observations, it is possible that H_2_O_2_ induces some higher-order modifications of p38, given the lack of recovery of full activity by DTT treatment in vitro (Figure 4). In contrast, since essentially all of the activity of the PGJ2 inhibited p38 is recovered by DTT treatment, PGJ2-induced oxidation of p38 appears to be completely reversible, suggesting lower order events at the dose range tested.

The use of PROP or similar approaches will allow protein oxidation reactions to be identified and quantified. Known targets of cysteine oxidation can be analyzed by immunoblot detection, similar to the approach used here for p38. In addition, since proteins are liberated with the target cysteines in their native state, the PROP procedure offers opportunities for proteomic studies to identify new protein targets of oxidation. We have performed pilot studies in which proteins from oxidized cells are recovered using PROP, released in DTT, labeled with iodoacetamide, and subjected to mass spectrometry analysis, with promising results.

While protein kinase activation usually involves phosphorylation of highly conserved serine, threonine or tyrosine residues within the “activation loop” of the protein kinase, the inhibitory redox switches that we and others have identified impact several divergent protein kinase regions. For example, MEKK1 is targeted by oxidation of a cysteine residue within the ATP binding domain [6]. PKA is oxidized within the activation loop in a manner that may regulate dephosphorylation of the activating threonine [28]. PKG1 is activated through oxidation to form a cysteine-paired dimer [7]. In other instances, several cysteine residues are oxidized, accompanying inactivation of the kinase, including JAK2, [9], and SAPK/JNK (Cross and Templeton, unpublished). In only a few instances have sites of oxidation been mapped to specific cysteines.

On the other hand, kinases that are related to one another likely share functional regulatory mechanisms, including the inhibitory redox switch. We identified the surface cysteines C119 and C162 of p38 as major sites of oxidation using mutant studies. On the linear subdomain map of p38 aligned with other protein kinases [29], C119 of p38 lies within subdomain V. Notably, p38 alpha and beta share a cysteine codon analogous to C119, while JNK1, 2, and 3 share a nearby cysteine in the same subdomain, and ERK isoforms lack cysteines in domain V. Cysteine C162, which is found in subdomain VIb, is shared among all p38 isoforms, and all JNK and ERK isoforms excepting ERK 3 and 4, but is otherwise lacking in other members of the CMGC kinase family (http://kinase.com/human/kinome/groups/cmgc.aln). This observation leads to the prediction that these conserved cysteine residues may serve to control other MAP kinase family members much as they do p38.

The ease by which specific cysteines and not others are oxidized may reflect the relative degree ionization of the target cysteine. Some oxidizable cysteines are also targets of electrophilic attack by drugs or signal-regulatory compounds originating outside the cell. For example the oxidized cysteine found in MEKK1 [6] is also the target of covalent alkylation with phenylethylisothiocyanate and sulforaphane, cancer chemopreventive compounds containing an electrophilic isothiocyanate group [30]. Other cysteines identified as oxidation sites thus represent potential sites of therapeutic regulation by drug candidates that may also covalently modify these target cysteines. Covalent targeting of regulatory cysteine residues is potentially the mechanism through which the protein kinase C (PKC) inhibitor bisindolyl maleimide (BIM) functions, since the maleimide moiety should make covalent bonds with target cysteines. While BIM is considered an ATP mimetic, we note that conventional and novel PKCs, that are inhibited by BIM, all have a cysteine residue within the ATP binding domain, while the atypical isoforms of PKC (iota and zeta), which are resistant to BIM, lack this cysteine. This appears not to have been explored in the literature.

The biological relevance of cysteine modification by electrophilic compounds such as oxidized lipids[31] and dietary isothiocyanates [30] raises the possibility that reversing the resulting Michael addition and thiocarbamate products (respectively) with DTT might result in identification of the targets of these reactions using PROP. Some evidence suggests that this is not so. For example, modification of the MEKK1 protein kinase by isothiocyanate is stable to boiling in SDS and DTT [30], though other cysteine adducts might be less stable. Similarly, numerous cell proteins labeled with biotin-prostaglandin J2, and detected with anti-biotin immunoblotting are not measurably reversed by boiling in SDS and DTT (Cross and Templeton, unpublished), though again, specific proteins might be. Owing to these observations, we predict that these reactions will not be detectable using PROP.

We [6] and others have previously detected regulatory oxidation of other protein kinases [7], [8], [9], [10], [11], [12], [13], [14], [15]. Importantly, in most of these experimental systems, oxidation is induced by hydrogen peroxide at high concentrations, diamide, menadione, or other non-physiologic agents. Our finding that micromolar concentrations of PGJ2 can induce oxidation and inhibition of p38 extends these results to agents involved in the physiologic response to disease or inflammation.

Our results suggest that oxidation may serve as a redox switch that provides a feedback mechanism for p38 and other signaling kinases. Specifically, initial activation of p38 results from phosphorylation within the “activation loop”, that is detectable using anti-phospho p38 antibodies. Subsequently, activated p38 may become oxidized at one or more cysteine residues, resulting in the loss of kinase activity. Importantly, *this inactive kinase retains its phosphorylation and may incorrectly be interpreted as an active kinase* when using anti-phospho antibody immunoblot as a surrogate measure of “activity”. Signal transduction investigators are cautioned that the simplicity of an anti-phospho-immunoblot may obscure more subtle means by which signal transduction is regulated. We recommend that for a specific signaling pathway to be deemed “activated”, a direct measurement of the catalytic activity in vitro is indicated, together with an evaluation of natural substrates of the kinase within cells.

## Materials and Methods

Cell culture, reagents and transfections. HeLa cells (American Type Culture Collection, ATCC.org) were grown in Dulbecco's Minimal Eagle's Medium supplemented with 10% calf serum and penicillin/streptomycin, in 5% CO_2_ environment. For experiments, cells were seeded in 6-well plates. Cells were transfected using Lipofectamine 2000 (Invitrogen, Carlsbad, CA) per manufacturer's instructions. Prostaglandin J2 (Cayman Chemicals, Ann Arbor, MI) was prepared in DMSO and stored in aliquots at −20°C. Antibodies used for immunoblotting were as follows: anti-p38 (Santa Cruz Biotechnology, Santa Cruz, CA), anti-phospho-p38 and anti-phospho-ATF2 (Cell Signaling, Danvers, MA), HRP conjugated goat anti-rabbit secondary antibody (Promega, Madison, WI). Blots were developed using enhanced chemiluminescence with images captured on a Fluor-Chem HD2 (Alpha-Innotech, San Leandro, CA) in 16 bit mode without image saturation. TIFF files were quantified using ImageJ (http://rsbweb.nih.gov/ij/).

p38 expression vectors. p38 was expressed from pcDNA3.1 containing a synthetic gene (GenScript, Piscataway, NJ) encoding murine p38alpha, isoform 1 (GI:10092590), tagged at the C terminus with a chitin binding domain. For the cysteine mutants, a second synthetic gene was designed that was identical to the first except that all of the cysteine codons were mutated to serine codons (4CS). To prepare the single cysteine add back mutants, restriction sites engineered between each of the cysteine codons were used to swap each individual cysteine codon into the 4CS mutant.

PROP procedure. Cells were rinsed quickly with HEPES-buffered saline and fixed in 10% TCA for 10 minutes to prevent post-lytic oxidation. Fixed cells on 35 mm wells were rinsed three times with methanol containing 10 mM N-ethylmaleimide (NEM), then air dried. Dry monolayers were dissolved in 0.25 ml of MLB/G (25 mM MOPS pH 7.1, 5 mM EDTA, 150 mM NaCL, 0.1% Igepal detergent, and 6 M guanidine HCL) containing 20 mM NEM. During a 2 hour total incubation period at room temperature, cells were scraped into microfuge tubes, and subjected to sonication (five ½ second pulses with a microtip probe) to shear DNA. Twenty percent of each sample was retained as a ‘Total’ fraction, and precipitated in 4 volumes of methanol. After the two hour incubation, DTT was added to 50 mM to reduce oxidized thiols, and incubated at 50°C for 30 minutes. Proteins were precipitated by addition of 4 volumes of methanol, with incubation for 2 hours or overnight at −20°C, followed by centrifugation at 2300×g for 5 minutes. Pellets were washed 5 times for 5 minutes each by rotational agitation in 100% methanol and recentrifugation, to remove all reducing agents. Drained pellets were redissolved in MLB/G containing activated thiopropyl agarose beads (GE Amersham, Piscataway, NJ) that had previously been washed rapidly in cold water per manufacturer's instructions, allowing 10 mg of dried beads per 35 mm well. Pellets were dislodged in this solution, and allowed to dissolve and react 2 hours or overnight with rotational agitation at RT, during which proteins with newly revealed thiols formed disulfide bonds with thiols on the beads. Beads were washed twice in MLB/G and three times in MLB (identical to MLB, without guanidine HCL). Drained beads, and ‘Total’ fractions, were resuspended in Laemmli sample buffer (2% SDS, 50 mM DTT, Tris•CL pH 8.8, 10% glycerol, and 0.01% bromophenol blue) and subjected to SDS-PAGE and immunoblotting. Gels were transferred to PVDF membranes and immunoblotted with anti-p38 antibodies. The protein purified on the thiol beads in the PROP procedure represents the oxidized protein present in the cells at the time of lysis, quantified as described above.

In vitro kinase assay. Cells were lysed in MLB and immunoprecipitated using immobilized anti-phospho-p38 conjugated beads (Cell Signaling). Beads were washed 3 times with MLB and once with 50 mM Tris (pH 7.4). p38 activity was assayed using 1 µg ATF2 substrate (Cell Signaling) in a 20 µL reaction containing 25 mM Tris (pH 7.4), 10 mM magnesium chloride, and 7.5 µM ATP. Samples were incubated for 30 minutes at room temperature, and then stopped by addition of 2X Laemmli sample buffer. Samples were separated by SDS-PAGE and phosphorylated ATF2 detected by immunoblotting.
